# Stakeholder-engaged research is necessary across the criminal-legal spectrum

**DOI:** 10.1017/cts.2022.501

**Published:** 2022-11-15

**Authors:** Alysse G. Wurcel, Christina Kraus, O’Dell Johnson, Nicholas D. Zaller, Bradley Ray, Anne C. Spaulding, Tara Flynn, Cynthia Quinn, Ronald Day, Matthew J. Akiyama, Brandon Del Pozo, Fred Meyer, Jason E. Glenn

**Affiliations:** 1 Department of Medicine, Division of Geographic Medicine and Infectious Diseases, Tufts Medical Center, Boston, MA, USA; 2 Tufts University Medical Student, JCOIN LEAP Scholar, Boston, MA, USA; 3 University of Arkansas for Medical Sciences, Little Rock, AR, USA; 4 RTI International, Division for Applied Justice Research, 3040 Cornwallis Road, Research Triangle Park, NC 27709, USA; 5 Associate Professor of Epidemiology, Rollins School of Public Health, Emory University, Atlanta, GA, USA; 6 Assistant Deputy Superintendent Health Services, Norfolk County Sheriff’s Office, Dedham, MA, USA; 7 Maricopa County Jail, Phoenix, AZ, USA; 8 The Fortune Society, Vice President of Programs and Research, Long Island City, New York, USA; 9 Department of Medicine, Montefiore Medical Center, Albert Einstein College of Medicine, Bronx, NY, USA; 10 Chief of Police, Ret., Burlington, VT, USA; 11 Deputy Chief (Retired), Las Vegas Metropolitan Police Department, Las Vegas, NV, USA; 12 Department of History and Philosophy of Medicine, University of Kansas Medical Center, Kansas, USA

**Keywords:** Jail, incarceration, stakeholder-engagement, prison, law enforcement, research ethics

## Abstract

People with lived experience of incarceration have higher rates of morbidity and mortality compared to people without history of incarceration. Research conducted unethically in prisons and jails led to increased scrutiny of research to ensure the needs of those studied are protected. One consequence of increased restrictions on research with criminal-legal involved populations is reluctance to engage in research evaluations of healthcare for people who are incarcerated and people who have lived experience of incarceration. Ethical research can be done in partnership with people with lived experience of incarceration and other key stakeholders and should be encouraged. In this article, we describe how stakeholder engagement can be accomplished in this setting, and further, how such engagement leads to impactful research that can be disseminated and implemented across disciplines and communities. The goal is to build trust across the spectrum of people who work, live in, or are impacted by the criminal-legal system, with the purpose of moving toward health equity.

## Introduction

Stakeholder-engagement in criminal-legal research is necessary to address health disparities for people impacted by the carceral system. The term “stakeholder engagement” was coined in parallel with patient-centered outcomes research (PCOR) [1] and is broadly defined as engaging people impacted by the healthcare system studied as equitable partners in research. Across the spectrum of criminal-legal settings and interactions – including but not limited to arrest, detention in jails, imprisonment, release, and court supervision in the community – people with criminal-legal system involvement have higher disease prevalence and mortality than people without such involvement [2–5]. Specific diseases, including mental illness and often inter-related substance use disorder, are highly prevalent in jailed and imprisoned populations [6–8]. As a result of the complex interplay between exposure to racism and racial violence, Black, Latinx, and Indigenous people are disproportionately incarcerated [9,10], and structural barriers prevent people with a history of criminal-legal involvement from accessing equitable healthcare upon return to the community [11–13]. Negative health outcomes are also experienced by people who work in the criminal-legal realm. Police officers and correctional officers are at increased risk of early mortality, hypothesized to be a result of occupational hazards and stress [14–18].

We are a coalition of clinicians, researchers, people with lived experience of incarceration, and people in law enforcement including in carceral settings, spiritual leaders, and advocates for criminal-legal and social justice reform who collectively write this paper as a call to action [4,19–33]. We have worked on research spanning methodologies including qualitative research, observational studies, quasi-experimental (natural experiments) studies, clinical trials, training initiatives, implementation research, and record-linking large administrative data sets in criminal-legal settings. After providing historical context, we will review barriers to research with people who are incarcerated, suggest solutions, and highlight successful strategies for stakeholder engagement.

## Historical and Contemporary Research Atrocities

It is critical to understand the legacy of unethical research on incarcerated people. Historically, the participation of incarcerated populations in biomedical research was often secured by combination of coercion and manipulation, including excessive payments and benefits, time away from the cell block interacting with medical professionals who were not as abusive as many correctional staff, and early parole consideration [34]. Enrolling in pellagra experiments at Rankin Prison Farm in Mississippi in the early 1900s, for example, was rewarded with early parole. Treatments for malaria [35], acne [36], and tularemia [37] were a few examples of the numerous medical advances developed through unethical research on detained and incarcerated people [38]. In a landmark 1968 study, professionals (e.g., doctors, lawyers) responded with more reluctance to participate in studies involving pathogens or toxins compared to prisoners [39]. The authors found that in addition to the undue influence of gaining social merit and financial incentives, the incarcerated persons expressed the opinion that participating in research elevated them to a protected level in the prison and connected them with doctors who cared about them. A particularly poignant line from a follow-up to the 1968 paper published by the authors in 1970 demonstrates this connection, “In part the research team has replaced the real family. Many prisoners would say, ‘I would do anything the doctor tells me to’” [40]. Dr. Albert Kligman, dermatologist, inventor of Retin-A acne medication, and lead researcher in the Holmesburg Prison, said “Many of the prisoners, for the first time in their lives, find themselves in the role of important human beings. We say to them, ‘You’re important, we need you!’” [36] The backbone of research in jails and prisons is based in the exploitation and manipulation as discussed above, and the available reports likely only capture a small percentage of the scope, breadth, and reach of unethical research done on people incarcerated in jails and prisons.

## Policy and Legal Changes for Protection from Unethical Research and Access to Ethical Research

Atrocities committed against people who are incarcerated in the name of research rightfully led to an overhaul of research ethics in the late 1970s to better ensure the ethical protection of vulnerable populations [36,41]. The implementation of these research protections led to a shift in biomedical practice during a time in which many social and cultural forces were beginning to culminate in nearly exclusive recruitment of white men for clinical trials [42]. Activism in response to the HIV/AIDS epidemic of the 1980s shifted the focus of research ethics from an emphasis solely on protection from harms to also improving access to research and its potential benefits. When done ethically, research improves healthcare. Research restrictions in the carceral setting prevented equitable access to emerging, life-saving treatments for HIV [43–46]. Experts in the field called for expanded access to ethically conducted correctional health research [47,48]. The 2006 Institute of Medicine delineated broad actions to expand research while continuing to protect people who are incarcerated [49].

Ethical research on the problems experienced by detained or imprisoned persons is not only possible in light of these considerations but also necessary for health equity. Despite these changes, people with criminal-legal experience continue to be under-represented and often systematically excluded from research, exacerbating health inequalities [50,51]. There is, in particular, a paucity of research on people who are in jails – a population that makes up most of the people who are incarcerated in the country [52]. Fear of repeating past exploitation and abuse fuels reluctance by academics, people with lived experience of incarceration, and carceral administrators to engage in research. Researchers should navigate conversations about the harms and inequities in these systems. A requirement for researchers doing so, however, is that they do not view people who are incarcerated through a paternalistic lens [53]. A degree of structural competency around issues of mass incarceration is necessary for all researchers who plan to conduct work in this space.

## Framework for Identifying Key Stakeholders

**Fig. 1.**
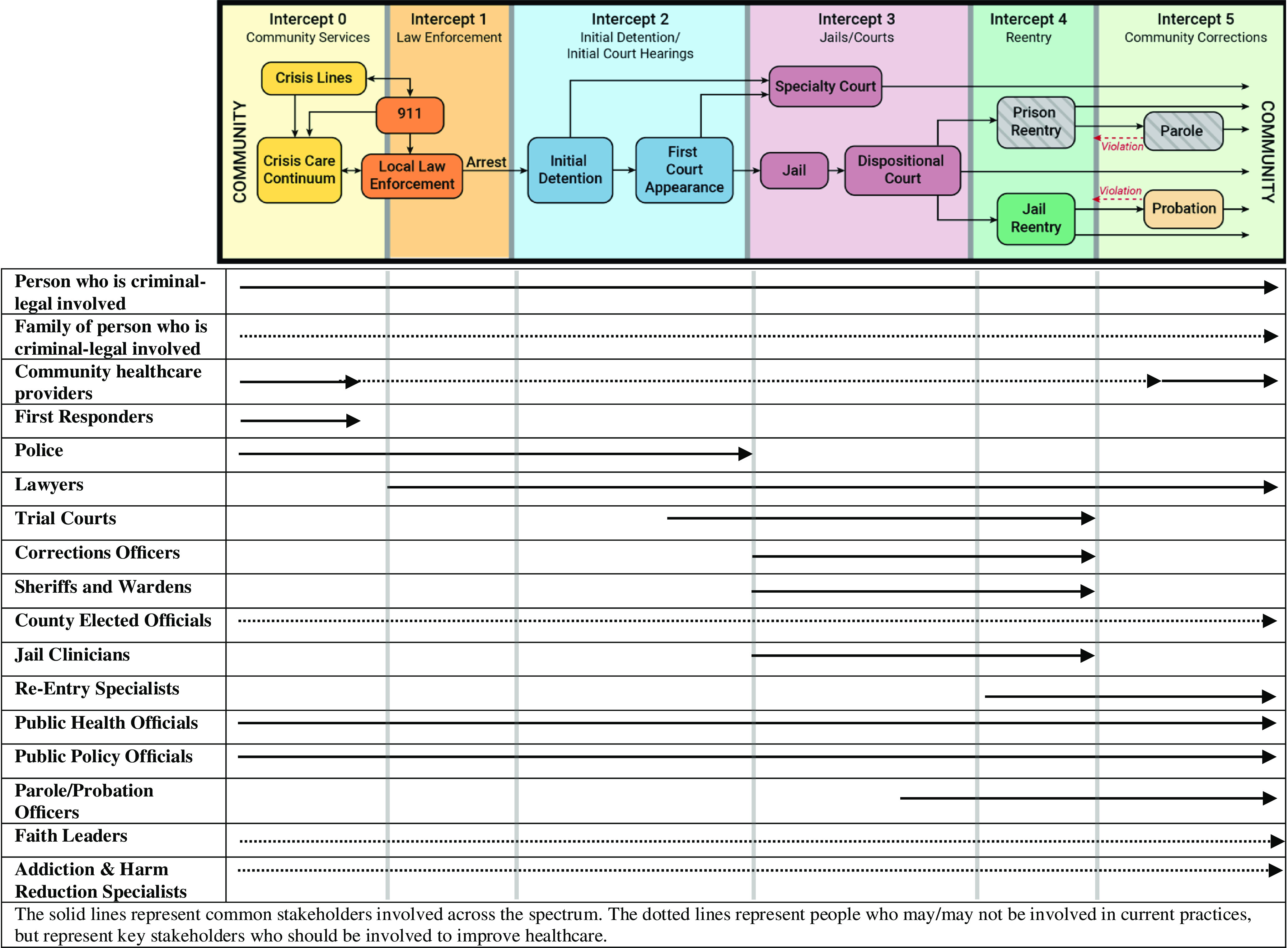
Sequential intercept model stakeholder engagement framework: we used the sequential intercept model (SIM) originally developed by Abreu D [54,55] to help identify stakeholders across the spectrum of criminal-legal involvement.

In Fig. 1, we use the sequential intercept model (SIM) as a framework for identifying important stakeholders to criminal-legal research [54]. We offer this model as a preliminary illustration to establish the contours of relevant populations and welcome the modification and improvement of this list to include as many peoples’ voices as possible. This model demonstrates the many dimensions within which to seek partners and serves as a reminder that there are many ways to develop a research team of stakeholders that touch each intercept collectively. At each step of the model, there are specific barriers and facilitators to engaging stakeholder groups as participants and collaborators in research. People with lived experience of incarceration, the only stakeholders who intimately experience every intercept of the SIM, are central and should be involved early and often. As Kara Nelson, a formerly incarcerated woman and Director of Public Relations and Development at True North Recovery, said, “We have to be at the table. We aren’t just redemption stories; we’re leaders who have something to say and something to offer, and we will be the ones with the solutions to make that change” [55].

The community where people who are incarcerated live and return includes crucial stakeholders. Non-engagement not only excludes these stakeholders from being a part of the solution, but it also allows for perpetuation of misconceptions, stigma, and discrimination in communities. Abrupt and cyclical transitions between community providers and jail clinicians disrupt the continuum of care, and community clinicians’ voices need to be heard in improving carceral health. Faith leaders in the community and in carceral settings are a part of a key group of stakeholders that, to date, have often been under-engaged by researchers. Many harm reduction, restorative justice, and treatment programs are also integral parts of the communities where many formerly incarcerated people seek care. All facets of the extensive legal system can have important insight into barriers and facilitators to improved healthcare delivery.

## Strategies for Engaging Stakeholders

As evidenced by increasing funding opportunities aimed at including people with lived experience of incarceration in the process of research, stakeholder engagement not only increases the likelihood of producing relevant research questions and successful interventions but also fosters lasting relationships that can be utilized over time as new challenges arise [56]. Several publications guide recruitment, engagement, and retention of stakeholders in research [57–59], outlining different timing (early on vs. continuous), organizational structures (advisory boards, working groups, consultants, participants), and remuneration (volunteer vs. paid). Here we focus on three groups of stakeholders: (1) people with lived experience of incarceration; (2) people who work in leadership positions in jails and prisons (e.g., sheriffs, superintendents, and wardens); and (3) people who work in trial courts, jails, prisons, and re-entry sites. In Table 1, we highlight studies that have successfully engaged these stakeholders in research, as well as other stakeholders across the spectrum of criminal-legal research.

**Table 1.** Summary of best practices for and lessons learned from implementation of stakeholder engagement by stakeholder group

Stakeholders	First author	Study summary	Key lessons learned
People who are incarcerated	Akiyama [92]	Qualitative interviews with people living with hepatitis C in New York City jails to better understand HCV treatment acceptability	Peers of incarcerated individuals served an important role in spreading HCV-related knowledge
Person who is criminal-legal involved Family/friend/caregiver of members of population Policy	Wennerstrom [63]	Overview of the “Prisoner to Patient project,” initiated in 2015 in New Orleans to develop a research agenda in tune with the health needs of people who were formerly incarcerated	(1) Iterative community engagement process: alternating community forum meetings with smaller advisory board meetings to evaluate whether proposed ideas were representative of person who is criminal-legal involved and their families’ needs. (2) “Getting on and off the bus” allowance: members of the research council could take breaks as needed from the project. If a participant took a break, one of the other stakeholders could contact them and troubleshoot barriers to engagement
Person who is criminal-legal involved and/or their family Clinicians Policy	Kendig [93]	Report on the experience of convening stakeholders of 27 different backgrounds, including trans people with history of incarceration, for a 2-day symposium in to gain consensus on barriers and facilitators to providing culturally competent, evidence-based care for incarcerated transgender people	Discussion across participants allowed for the development of “consensus considerations”: (1) identifying correctional policies for people who are transgender during incarceration that protects them from abuse and creates a culture of safety, (2) training correctional staff to enhance respectful attitudes toward trans inmates and coworkers, (3) better defining medically necessary care and improving access, and (4) identifying effective re-entry strategies for transgender persons. Symposium participants, many of them leaders in their field, are also able to use the insight gained from this session in the regions/facilities where they practice
First responders	Del Pozo [94]	Analysis of a trial online training for education aimed at reducing false beliefs about fentanyl. Comparison between baseline and follow-up assessment showed reduction in false beliefs about fentanyl	Partnering with first responders can identify and correct points of misinformation. Training on the potential for fentanyl overdose during police encounters can improve the public health response to the overdose crisis
Trial courts Justice-involved women	Roth [95]	Researchers utilized community-based participatory research (CBPR) methods to engage justice-involved women and court staff in the development of sexual health services	CBPR methodology was effectively used in the court-setting to develop research questions about current gaps in services and identify opportunities to improve a court-based screening program
Corrections officers Union leadership	Namazi [96]	Cross-disciplinary collaboration to develop and implement a peer health mentoring program for correctional officers (The Health Improvement through Employee Control Study)	Power sharing between researcher and correctional officer (CO) was important. Separate meetings were held for (1) researchers and union leadership and (2) correctional officers. Information from meetings was reported back to inform group (1). Having separate space for meetings empowered CO participants
Corrections officers People with lived experience of incarceration	Khorasani [77]	Institutional surveys were deployed to inform vaccine operationalization at Middlesex County Jail; surveys were analyzed secondarily as research	Although the majority of both correctional officers and people incarcerated in jails were interested in receiving the COVID-19 vaccine, several people expressed concerns about vaccine safety. Partnering with correctional leadership allowed this information to be utilized in vaccine rollout
Jail clinicians Students	Ekaireb [97]	Interviews with carceral health clinicians to understand provider knowledge about advanced care planning (ACP) in prisons and jails	Researchers outlined direct policy interventions that were informed by the clinicians that will implement them. Examples include initiating provider training in ACP and revising institutional policy so that all patients can receive ACP (rather than just critically ill)
	Hashmi [31]	Researchers spoke with medical learners working in a correctional facility that was partnered with an academic medical center for healthcare services for people experiencing incarceration	This study points to the potential harms of partnerships with academic medical centers and the need for intentional preventive measures such as improved training for healthcare providers at all levels
Re-entry specialists People with lived experience of incarceration	Victor [20]	Analysis of the effectiveness of substance use programming for person-oriented recovery and treatment during the time of re-entry; provides guidance on involving peer workers as mentors	Peer recovery coaches provided feedback on the research study and influenced decisions on protocol modifications such as incentive structures and data collection plans
Jail administrators People who are incarcerated Community clinicians	Evans [98]	Description of the goals of the Massachusetts Justice Community Opioid Innovation Network, an initiative connecting academic partners, community clinicians, and jail administrators to conduct implementation science research around expanding delivery of medications for opioid use disorder in MA jails	The paper details the development of mixed methods research tools to evaluate current mechanisms of treating opioid use disorder and measuring implementation and efficacy outcomes. There is discussion of how the researchers adapted research activities in response to the COVID-19 pandemic
Parole/probation officers	Brogan [82]	Researches engaged probation officers in the improvement of juvenile probation by utilizing community-based participatory action research principles to build a training	Before initiating a training, the probation officers were interviewed and the team determined potential points of resistance from the officers. This allowed for explicit resolution of concerns and impacted the success of the training
Faith leaders Sheriffs People who are incarcerated Faith leaders Community clinicians	Crist [99]	This paper shared lessons learned from community, faith-based action research with a group of formerly incarcerated women in southern California	Researchers, faith leaders, and community members collectively reflect on research experiences to determine findings and ensure mutual learning
	Erfani [22]	With approval from several of the Massachusetts county sheriffs, clinicians from the community, medical students and faith leaders went to jails to educate about COVID-19 vaccination in a format called “Ask Me Anything”	Participation in clinical counseling, in partnership with community leaders, in in-person communication sessions in housing units. Specifically, session leaders built trust during these sessions by acknowledging systemic racism, medical abuse, and individuals’ agency in the vaccination decision process
Addiction specialists Juvenile carceral center staff	Johnson-Kwochka [100]	This research paper details a study built on the collaboration between community mental health centers and juvenile justice centers for adolescent substance use disorder treatment	Researchers used the adopter-based innovation model to find commonalities between the process of evidence-based project implementation within community mental health and juvenile justice centers and found that they had similar shared perceptions regarding substance use that could facilitate collaboration
Clinicians Corrections administrators	Lee [76]	A survey of US correctional facilities was used to identify barriers and facilitators to receiving H1N1 influenza vaccine during the pandemic. The study found that 57% of smaller jails surveyed never received vaccines	This study informs how more robust partnerships between public health entities and correctional facilities will improve emergency response systems to threats such as the influenza pandemic. Direct communication between these groups can limit confusion and streamline protocol initiation
Public health officials Police	Goulka [75]	This paper details the goal of re-envisioning police reform through the lens of public health	Partnering and training public safety officers/leaders to adopt public health metrics for reform. These metrics would also be guided by evidence and informed by the community members they serve

### People with Lived Experience of Incarceration

People who are incarcerated may be reluctant to participate in research for many reasons including (1) fear of differential treatment and other safety concerns relating to reactions from carceral staff; (2) discomfort disclosing personal or health information; or (3) stigmatization/negative response from family members and peers [60]. Through the process of Institutional Review Board (IRB) submission (discussed below), there are checks and balances in place to guard against coercive research. In addition to the IRB, however, it is the researcher’s job to think critically about any ways in which the research may be coercive. As another safeguard against unethical practices, people with lived experience of incarceration should not only be asked to participate in research but also involved in the development of research ideas, oversight of the research, and publication and dissemination of the results. As involved with the criminal-legal system as some administrators and employees are, without the input of those who most thoroughly understand the failures of the carceral system, research will fall short of its aims [61].

Partnering with community-based organizations focusing on decarceration and empowering people with lived experience of incarceration, such as The Fortune Society and Just Leadership USA, may be one way to ensure that research topics reflect the concerns of people with lived experience of incarceration. Collectively, in our practices, and in the present body of research created by partnerships with people with a lived experience of incarceration, we have found that employing principles of community-based participatory research (CBPR) and PCOR is vital to inclusive research efforts when appropriately tailored to the context [62]. To ensure participation by persons with lived experience in research is consistent, a member of the research team may be assigned to make periodic, supportive check-ins with team members throughout the research period [63]. As detailed by the experience of Wennerstrom et al., failure to do so can preclude their ability to balance the struggle of re-entry into community and participation in a project and can be avoided by using an “on and off the bus allowance” (see Table 1). One example of how to set a research agenda with CBPR is the Prison Research and Innovation Initiative of the Urban Institute. Their work with stakeholders in Colorado, Delaware, Iowa, Missouri, and Vermont demonstrates how incorporating the insight of incarcerated individuals yields more credible research and projects that go on to produce more useful findings that contribute to reform [64]. Another example is research by Victor et al., in which peer recovery coaches (PRCs) in a substance use recovery program for returning citizens were the drivers of protocol reform for a clinical trial [20]. The involvement of PRCs led to more useful data collection that went on to be used for improvement of this important re-entry program.

Exposure to incarceration is linked to negative health outcomes, and engaging people with history of previous incarceration is important to develop improved systems of care [11,65,66]. Outside of recruiting from community supervision sites (e.g., parole and probation offices), it may be difficult to identify people with lived history of incarceration. The electronic health record captures important data points which can be queried to develop research cohorts, but history of incarceration is not systematically included. People with experience of incarceration may be reluctant to report this to clinicians for fear of being subjected to stigmatizing views or receiving suboptimal care, which could potentially delay diagnosis of illness and treatment of pain. Ideally, clinicians will ask about a history of incarceration in order to better deliver culturally competent, trauma-informed care and adapt to the specific needs of people who have experienced incarceration [67–69]. The development of local, institutional, and national systems to identify people with lived experience of incarceration who are interested in participating in research is one tangible action item that could help facilitate impactful research aimed at improving healthcare delivery. Researchers should go to the communities where people with lived experience of incarceration live and bring the research to them. Increasing accessibility may also mean having locations close to public transit, reimbursing for transportation, and allowing people to bring children to research visits.

### People in Jail and Prison Leadership Positions

Building trusting relationships with people who are in administrative positions overseeing jails and prison takes time and an open-minded attitude to learn about the challenges faced by correctional administrators. Carceral settings, police departments, and trial courts are complex systems comprised of relationships and hierarchy, which may not always have the same intents and priorities as researchers [70,71]. Researchers should be aware of formal and informal gatekeepers who pose barriers to research; these might be organizations or persons, sometimes those in charge of agencies, with the power to open or withhold access [72]. Knowing the gatekeepers, and how they are perceived by other stakeholders, can play an important role in rapport building [73].

It will often take time to build trust with leadership of jails and prisons who may have had negative experiences with researchers in the past. Establishing oneself as a “trusted outsider with insider knowledge” can be an effective way to gain trust and access for many researchers [74]. While norms toward virtual meetings have shifted because of the COVID-19 pandemic, public safety work is often hands-on, and meeting in-person can overcome sociocultural barriers. Attending local, regional, and national correctional conferences (e.g., National Commission on Correctional Health Care, the National Sheriffs’ Association, and the Academic Consortium on Criminal Justice Health) can connect researchers with administrative leaders in the field and facilitate one-on-one face time vital for building trust. Connecting leadership from these groups with public health agencies in more formal relationship building will also allow for more streamlined communication in case of emergency (as seen with constantly adapting COVID-19 policies) and further will allow for more upstream overarching changes to the structurally violent carceral system as a whole. Some research initiatives lead by authors like Goulka [75] and Lee [76] have begun this work (see Table 1) by demonstrating the untapped benefits of such relationships and represent an impetus for further work to convert these often dichotomous agencies into a more unified entity.

### People Who Work in Carceral Spaces and Law Enforcement

People working in law enforcement, including jails and prisons, have important insight on topics such as vaccination, solitary confinement, and women’s health [77–79]. Common concerns from discussions about enrolling people who work in carceral spaces in research include (1) potential workplace stipulations barring employee participation in research; (2) confusion about whether people working in correctional settings can take stipends in return for research participation; and (3) employee concern that participation in research may be reported to leadership and used as grounds for discipline, termination, or ostracization. Inviting people who work in the criminal-legal system to participate on self-identified issues in jail and prison culture improves health for both residents and staff [80]. Seeking their perspective will likely build support for the broader research endeavor [20]. Officers provide feedback to researchers for successful study implementation; they can identify organizational and cultural barriers and offer workable solutions [81]. An example of how this engagement can be navigated and lead to improved study outcomes is seen in the success of a community-based, participatory action research-guided training program that facilitated probation staff individual attitude and practice changes for the improvement of juvenile probation case management. These positive outcomes and changes were able to prevail despite organizational, cultural barriers (see Table 1) [82].

Innovative ideas on how to engage people who work in jails to help support a culture of quality improvement and research in the jails and prisons need further consideration. One potential idea is to create a national certificate program for corrections officers with education about the history of research in carceral spaces, best practices for research, and opportunities to be mentored in the development of research projects. Part of this training could include workshops that facilitate communication between carceral staff and those experiencing incarceration, breaking down a historically prominent barrier for the achievement of the common goals of (1) supporting both groups as researchers, learners, and leaders and (2) improved research outcomes. Correctional officers are a population at risk for early mortality and are overall understudied as an occupation with potentially high job-related risks [83–85]. Training corrections officers on the importance of research to improve outcomes for everyone, not just people who are incarcerated, should be imbedded in any program about research in jails and prisons.

## Planning for the IRB Review

Once gatekeepers have authorized and support research, the next step for the researcher is gaining IRB approval. Conducting research to better understand the structural and systematic aspects of health and healthcare in carceral settings finds strong ethical footing. However, the IRB approval process can be challenging. Many IRBs require a letter of support, even for non-human subject research, from executive leadership at carceral institutions. Federal regulation, encapsulated in 45CFR46 Part C, imposes specific provisions for IRBs when research involves people who are incarcerated. For instance, IRBs must have a “prisoner representative,” who provides an extra step of review for any research related to people who are incarcerated. Some institutions facilitate meetings between the research team and IRB staff to discuss the research protocols prior to submission and to help identify points that should be highlighted or clarified. Challenging areas include confidentiality and coercion/compensation. Some carceral settings allow audio-recording, while others do not. The use of technology such as smart phones, tablets, and computers is generally restricted for security reasons. Detailed consultation with both the correctional facility and persons who have experienced loss of liberty prior to finalizing a protocol can prevent problems later. Each carceral site has their own set of policies and procedures for participant reimbursement. Some jails and prisons allow for money to be deposited into a person’s commissary fund – money they can use to buy food or personal hygiene items – and some settings allow for the money to be placed in their personal property that they will receive upon release. However, because many incarcerated persons are not free to earn other sources of income, past exploitative research practices on incarcerated persons revealed that even minor reimbursements are often coercive. This tension between the goal of fairness and the goal of protection is one not easily resolved while working within the confines of the carceral system [86,87].

## Finding Funding

Funding for correctional health research is limited and disproportionate to the size of the US correctional population [88]. There has been progress, with large initiatives like the Justice Community Opioid Innovation Network [89] and a National Institutes of Health (NIH) program [90] that awarded more than $100 million to-date toward investigating gaps in opioid use disorder (OUD) treatment experienced by people in criminal-legal systems. Most people with OUD will have some degree of involvement with these systems in their lifetime, making the need for such funding to correct disproportionate disease burden staggering [91]. The investment, however, is limited to the study of one disease process and is insufficient considering the totality of funding needed to address the significant health inequities faced by incarcerated populations. Additionally, as research on the topic of incarceration does not neatly fall into the scope of NIH institute scientific plans, it can be challenging to find grant reviewers with topical and methodological expertise. In addition to earmarked national funds used for research aimed to improve healthcare for people who are incarcerated and with lived experience of incarceration, increasing access to philanthropic and foundational grants for researchers will help fuel the pipeline of research.

## Conclusion

Working from a legacy of unethical research with deep roots, the future of research in the criminal-legal realm must be rebuilt on a foundation of trust between all stakeholders. The COVID-19 pandemic galvanized successful cross-disciplinary relationships between public health, academia, and correctional administrators to address the substantial burden of COVID-19-related morbidity and mortality within carceral settings. Now is the time to cultivate the seeds of this nascent collaboration. Engagement of diverse stakeholders in equitable and rigorous research will help to mitigate health inequities that are all too common in carceral settings. Formerly incarcerated people should be involved in the organizational structures to bring voice to their lived experiences as it relates to healthcare while incarcerated and access to healthcare after release. In conjunction with structural and policy changes aimed at decarceration and health equity, these research initiatives stand to improve the health of people and communities exposed to the carceral system. We write this manuscript to encourage our colleagues to find partners with lived experience of incarceration and working in criminal-legal settings and involve them in identifying research questions and collaborating in the research process as a critical step toward improving healthcare equity.
